# Impact of the Surface Irregularities of NiFeMo Compacted Powder Particles on Irreversible Magnetization Processes

**DOI:** 10.3390/ma15248937

**Published:** 2022-12-14

**Authors:** Denisa Olekšáková, Peter Kollár, Miroslav Neslušan, Miloš Jakubčin, Ján Füzer, Radovan Bureš, Mária Fáberová

**Affiliations:** 1 Institute of Manufacturing Management, Faculty of Manufacturing Technologies, Technical University of Košice, Bayerova 1, 08001 Presov, Slovakia; 2 Institute of Physics, Faculty of Science, Pavol Jozef Šafárik University in Košice, Park Angelinum 9, 04154 Kosice, Slovakia; 3 Faculty of Mechanical Engineering, University of Žilina, Univerzitná 1, 01026 Zilina, Slovakia; 4 Institute of Materials Research, Slovak Academy of Sciences, Watsonova 47, 04001 Kosice, Slovakia

**Keywords:** magnetic materials, permeability, supermalloy, compacted powder materials

## Abstract

One specific group of materials with excellent application potential are powder-compacted soft magnetic materials. These materials have been intensively studied by materials scientists to improve their magnetic properties. This work describes the influence of mechanical smoothing applied to Ni80Fe15Mo5 (wt.%) alloy particle surfaces before the process of compaction. The soft magnetic properties of compacted powders prepared from smoothed and non-smoothed particles were investigated using the following measurements: coercive field, permeability, excess loss, and Barkhausen noise analysis. We found that compactions prepared with smoothed powder particles exhibit a lower value of coercivity (4.80 A/m), higher initial (10,850) and maximum relative permeability (27,700), and low-frequency core losses (1.54 J/m^3^) in comparison with compactions prepared with non-smoothed particles.

## 1. Introduction

Due to the worsening situation regarding the world’s energy supply, many corporations are looking for new materials, as well as new preparations and solutions for them, aimed at reducing energy losses as much as possible [1,2]. Therefore, specialists in materials research who deal with material compositions and their properties are also focusing their attention on the method of preparation and subsequent treatment of such materials [3]. Magnetic materials are important in industry, materials engineering, energy, and automotives, as well as other industries where electronic and electromechanical devices are developed and manufactured [4]. Important members of the magnetic materials group are soft magnetic materials, which possess interesting properties, such as low values of coercivity, low values of hysteresis loss, and high values of permeability. In modern material research, the intervals of the magnetic properties of soft magnetic materials are still at the foreground [5]. Soft magnetic compacted materials prepared with compacted powders are used as components in electromotors, magnetic circuits, cores, and computers, among other things [1,5].

Soft magnetic metal materials in the form of 3D bulk, prepared by compacting powder particles, have high permeability, lower magnetostrictive coefficients, and high magnetic flux densities of saturation. The disadvantages of these materials are higher values of coercivity and core losses [6]. Nowadays, useful soft magnetic metal materials (Fe-based) include Fe, FeNi (called permalloys), FeSiAl, FeSiCr, and FeNiMo (called supermalloys), among others. They exhibit good mechanical properties and can be produced at relatively low production costs [7,8]. The addition of molybdenum to these alloys leads to higher permeability and lower eddy current loss than in other soft magnetic metal materials, so alloys such as FeNiMo can be used at higher operating frequencies [9]. One of the ways to improve the magnetic properties of FeNiMo is an innovative method involving the preparation of powder particles, which are used for compaction. We can improve the properties of soft magnetic moldings by modifying the surface of the powder particles from which they are prepared, the so-called smoothing process. This process helps improve the magnetization processes taking place in these small powder particles as they interact with other surrounding particles [10]. The present work explains the influence of interactions between powder FeNiMo particles (with non-smoothed particle surfaces and smoothed particle surfaces), which are part of compacted materials, based on two complementary approaches to the analysis of their structure and magnetic properties.

Powder particles are modified during the smoothing process [8,11], and this condition significantly affects them. During smoothing, surface irregularities are removed on the surface of particles, which prevents the displacement of their domain walls. During mechanical milling, smoothing and compacting can cause structural defects, and these can have a negative impact on the domain-wall displacement processes [12,13]. For this reason, we used Ni80Fe15Mo5 alloy for our research, for which the value of magnetostriction is close to zero and magnetostriction induces stress fields with only a limited negative effect on the displacement of the domain wall [14,15].

The present work describes an explanation of the influence of the interaction between powder FeNiMo non-smoothed particle surfaces and smoothed particle surfaces. These mechanically prepared particles were part of the compacted material and were based on two complementary approaches to analyzing their structures and magnetic properties. The compaction prepared with smoothed powder particles exhibited better soft magnetic properties (lower coercivity, higher initial and maximum relative permeability, and low-frequency core losses) in comparison with the compaction prepared in the same conditions with non-smoothed particles. The positive influence of the smoothing process applied to the particle surface on the soft magnetic properties can be explained by the reduction in the number of surface obstacles for the domain wall’s displacement.

## 2. Materials and Methods

For our experiments, we chose the polycrystalline material Ni80Fe15Mo5 (wt.%), which exhibits near-to-zero magnetostriction in terms of eliminating the other influences of the surface treatment, except in terms of its influence on the residual shape of the powder particle element.

**Figure 1:**
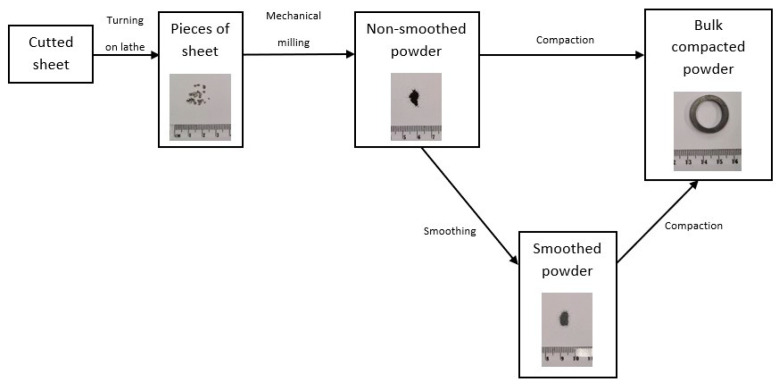
The preparation route of Ni80Fe15Mo5 compacts.

**Figure 2:**
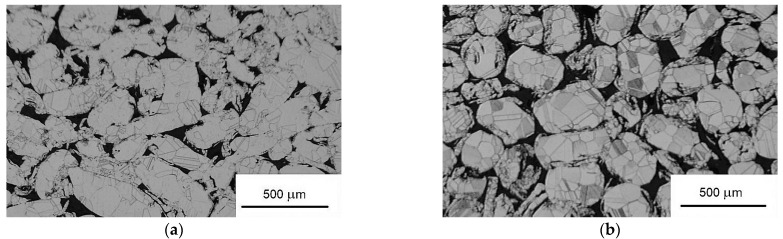
The morphology of (**a**) sample N, (**b**) sample S.

Two samples (Figure 1) of soft magnetic compacted powder with the chemical composition Ni80Fe15Mo5 (wt.%) were prepared for further investigation, according to our previous experiences [8,10,11,14]. Small chips (1–5 mm) were prepared by dry grinding a sheet with a thickness of 0.6 mm using the rotary drill of a lathe. These chips were mechanically milled in a Retsch PM100 planetary ball mill at room temperature. We used a steel vial and steel balls for milling. Mechanical milling was performed for 3 hours with a BPR of 9:1 at 300 revolutions per minute. The milling process was carried out intermittently every 2 min. The direction of the rotation was reversed. The resulting powders were sieved to obtain the required size fraction (100–300 µm), and their elements showed an irregular shape; see Figure 2.

The first sample—“sample N” (Figure 2a)—was prepared via the compaction of powder (without any coating) with a pressure of 700 MPa (uniaxial) at a temperature of 410 °C for 5 min. Then, “sample S” (second sample) was prepared via pressing under the same conditions, but the particles of powder were smoothed before the compacting process took place (Figure 2b) using a mechanical treatment process. This led to smoothing of the edges of irregularities on the surfaces of the milled powder elements, which was executed in a 1000 mL vial on the planetary ball mill without balls, with abrasive paper glued to the inner cylinder shell (the mean diameter of the grit was 10 µm). The weight of the powder that was mechanically treated inside the vial was 20 g. The treatment process was executed for 20 min, the angular velocity was 500 rpm, and every 120 s, the direction of rotation was changed [8]. After the smoothing process, the particles of powder were magnetically separated. Element analysis of the control was performed via SEM and it did not show the presence of contaminants [8,11]. After compaction, both compacts were sintered in a hydrogen atmosphere as follows: the samples were rapidly heated up to 1100 °C at a heating rate of 10 °C/min; after the hold-up period, which was 6 h, the samples were rapidly cooled to 300 °C at a cooling rate of 10 °C/min, and then, they were taken out of the furnace. As presented in [11], the prepared compacted samples of powder particles, when the surface irregularities of the particles were partially removed, had better soft magnetic properties.

The graphs present hysteresis loops and permeabilities in the internal magnetic field when the external magnetic field is corrected, with the demagnetizing field based on the experimental values of the demagnetizing factor [8].

The prepared compacted ring-shaped samples (Figure 1) were used for further investigation of the structure and magnetic properties (the measurement of hysteresis loops, coercivity, permeability, etc.). Their dimensions were as follows: the external diameter was 24 mm, the internal diameter was 18 mm, and the height was 2.9 mm.

## 3. Results and Discussion

### 3.1. Microstructure

The microstructure of the surface (polished and etched) of both compacted powder samples (sample N and sample S) was investigated using a Nikon Eclipse LV DIA optical microscope using etching via Marble’s reagent [16] (see Figure 2). The shapes of and the differences between the non-smoothed powdered particles (Figure 2a) and smoothed particles (Figure 2b) are significant and visible. We can observe that sample N typically has elongated particles of irregular shapes, and the powdered elements of sample S with smoothed particles show more spherical particles with fewer sharp edges. It seems that the deforming bands on the particles with smoothed surfaces are denser and thinner, and the increase in the band spacing is induced by higher compressive deformation in comparison with particles without smoothed surfaces [17]. During powder compaction, a deformed structure of the ferromagnetic element of both samples is observed. It is mainly characterized by the existence of deformation of the shear band due to the pressure between powder particles during compaction [18]. Due to the irregular shape of the crack part of sample N, there is better contact between the particles; howeveer, this phenomenon is not dominated by the fact that magnetization processes take place more easily in the particles of sample S, where the capture centers for the displacement of the domain walls are reduced. Due to the near-to-zero magnetostriction of the used permalloy’s composition, only a negligible effect of the defects induced by the surface smoothing process on domain-wall displacement can be expected [19].

The density of sample N prepared from non-smoothed particles is 7.30 g/cm^3^. The density of sample S prepared from smoothed particles is 7.15 g/cm^3^. These values were calculated from the dimensions and the weight of the samples, are slightly lower than those of the bulk material of the same chemical composition in an as-cast state (8.83 g/cm^3^) due to the presence of air pores in the compactions [20]. The density of sample N is closer to the theoretical density of the material because, while the particles are non-smoothing, they are cracked in the process of non-compaction. Small pieces of the cracked particles fill the space better than in sample S (there is less particle breakage).

The density values of both samples are comparable. The density of sample N prepared from non-smoothed particles is higher compared to the density of sample S prepared from smoothed particles, assuming breakage of the non-smoothed powder particles and the generation of small pieces of ferromagnetic material filling the cavities between large particles. These angular and protruding parts are exposed to higher pressures, which have a negative effect on the structure of the material (many structural defects such as additive surface edges and dislocations, which partially hinder the displacement of the domain walls). The structural defects cause degradation of soft magnetic material properties. The gaps between the particles of the smoothed powders of sample S are smaller and have lower values of density. Thus, we assume that the pressure that acts on the particles during the pressing will be evenly distributed over the entire surface and its distribution will be more homogeneous. The smoothed powder particles of sample S have partially removed surface obstacles for domain-wall displacement, resulting in better soft magnetic properties of the materials in accordance with sample N prepared from non-smoothed particles [8].

### 3.2. Coercive Field

**Figure 3:**
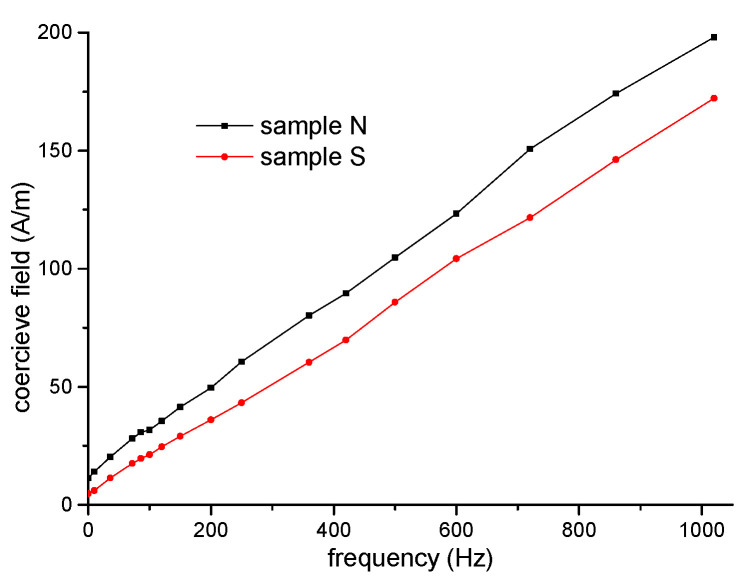
The values of the coercive field in frequency range from DC to 1 kHz for both samples.

The coercive field was measured using a hysteresis graph at a maximum induction of 0.2 T in a frequency range from DC to 10 kHz (see Figure 3).

Therefore, the increase in the coercive field with the frequency of sample S, prepared through the compaction of powder (with smoothed powder particles), exhibits lower values of core losses and the coercive field than sample N prepared via the compaction of non-smoothed powder particles.

### 3.3. Hysteresis Loops

**Figure 4:**
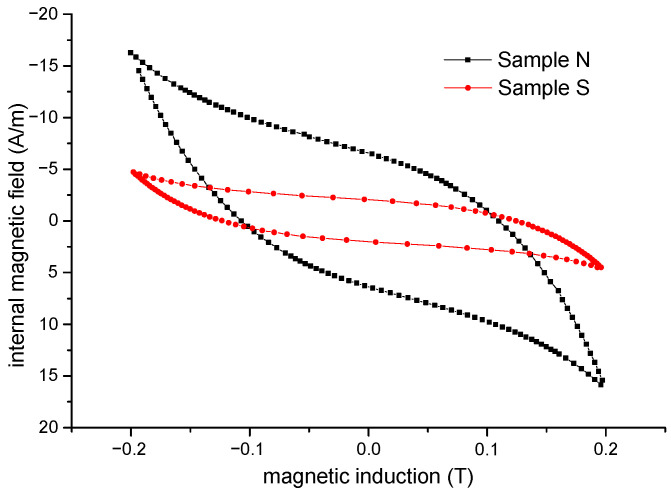
Hysteresis loops of sample N and sample S (rotated at a 45° angle towards the left).

To investigate the magnetic properties, such as the coercive field and permeability, the hysteresis loops (at a maximum induction of 0.2 T) of both samples were measured via DC hysteresis graph using a fluxmeter [21]. As you can see in Figure 4, the graph of the hysteresis loops for both samples has been rotated at a 45° angle towards the left to obtain induction B on the horizontal axis for better presentation of the other quantities as a function of B.

We can see that the value of the coercive field is lower for sample S. This is probably due to the changes in the dynamics of the magnetization process in sample S in comparison with sample N, where the edges of the particle surfaces act as obstacles for domain-wall displacement.

### 3.4. Permeability

Magnetic permeability *μ* is one of the characteristics of the magnetic properties of a material or a property of a given material or medium that quantifies its magnetic response of induction *B* when subjected to magnetic field strength *H*. Magnetic permeability can be defined depending on the characteristics of the changes in *B* and *H*. We know many definitions of this property, but generally, permeability is characterized as a proportion of the magnetic field to the magnetic induction.

The total relative magnetic permeability *µ_tot_* is defined as follows:

(1) $$\mu_{tot}=\frac{B}{\mu_{0}H\;},$$

where *µ*_0_ is a magnetic constant of free space.

It is more appropriate to use the calculation of the total permeability according to equation (1) to analyze the magnetization process along the initial magnetization curve, but it is not suitable to apply it along the hysteresis loop. The dependence of *μ_tot_* = f(*H*) allows us to calculate the initial and maximum relative permeability. The values are presented in Table 1.

The derivation of the initial magnetization curve or hysteresis loop at each its point [*H_i_*, *B_i_*] is called differential relative permeability *µ_diff_* and it shows the response of the material (system) to the changes in the magnetic field.

(2) $$\mu_{diff}=\frac{1}{\mu_{0}}{\left(\frac{dB}{dH}\right)}_{H_{i},B_{i}}$$

This approach is suitable to describe magnetization processes as domain-wall displacements and rotations of magnetization vectors and their contribution to the magnetization process inside a magnetic material, and these processes may be reversible or irreversible. In 3D soft magnetic materials, the most common reversible processes are reversible domain-wall displacement and reversible magnetization vector rotation. The dominating irreversible process is irreversible domain-wall displacement caused by Barkhausen jumps. Irreversible magnetization vector rotation is not common in bulk soft magnetic materials [20].

**Figure 5:**
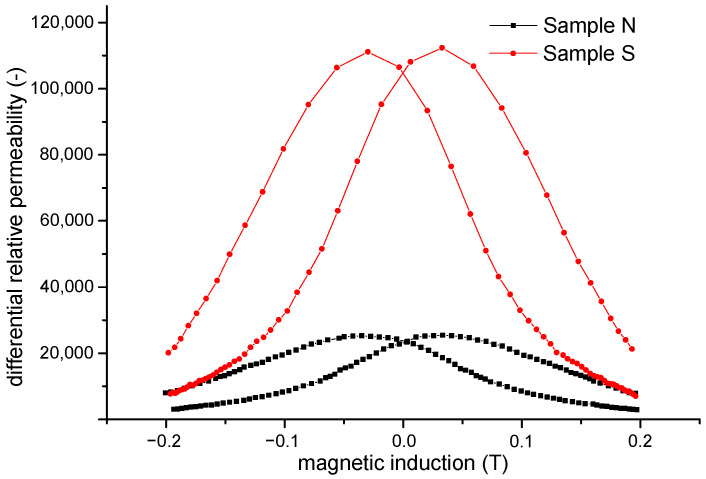
Differential relative permeability of sample N and sample S as a function of induction.

The differential relative permeability along DC hysteresis loops for both samples was obtained from the measured hysteresis loops (Figure 4) up to a maximum induction of 0.2 T. Figure 5 shows the values of differential permeability *µ_diff_* for both samples as a function of induction.

The value of differential permeability for bulk sample S is higher than that for sample N. This is in accordance with the measured values of the initial and maximum permeability (see Table 1) and the coercive field (see Figure 3), confirming the assumption that in sample N, the surface irregularities act as domain-wall obstacles for domain-wall displacement, leading to a general decrease in differential relative permeability.

The reversible relative permeability *µ_rev_* is measured in the same way as the incremental value, but with the amplitude of the AC excitation as small as practically possible (*H* → 0). The term “reversible” implies that the process is lossless. Additionally, it is the case for the initial permeability.

(3) $$\mu_{rev}=\frac{1}{\mu_{0}}\underset{\Delta H\to 0}{\lim}{\left(\frac{dB}{dH}\right)}_{H_{i},B_{i}}$$

**Figure 6:**
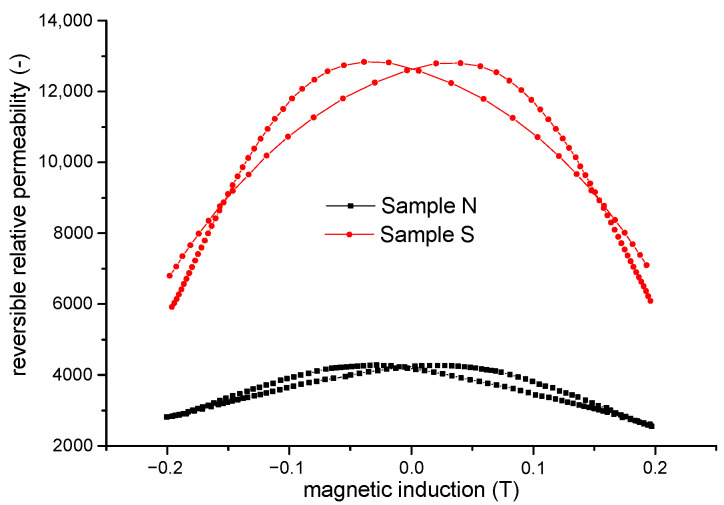
Reversible relative permeability of sample N and sample S as a function of induction.

Figure 6 shows the measured reversible permeability as a function of magnetic induction for sample N and sample S.

We can see that sample S exhibits higher values of reversible permeability. Consequently, the domain wall of this sample is more movable.

The irreversible relative permeability *µ_ir_* is influenced by irreversible magnetization processes (mostly by irreversible domain-wall displacement) and is calculated as the difference between the differential and the reversible relative permeability.

(4) $$\mu_{irr}=\mu_{diff}-\mu_{rev}$$

**Figure 7:**
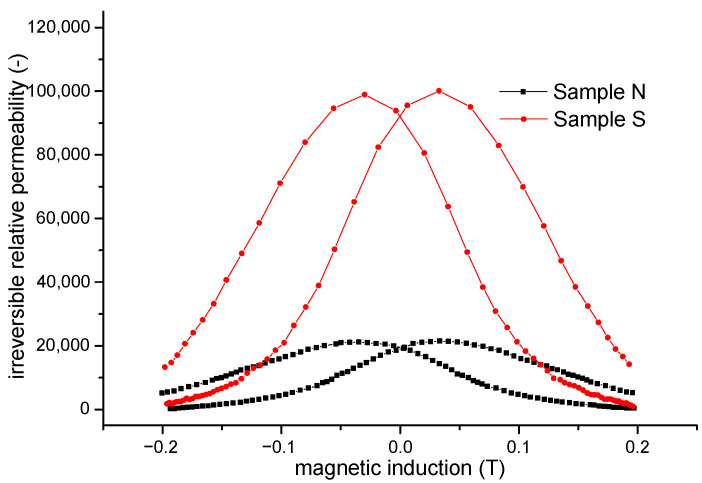
Irreversible relative permeability of sample N and sample S as a function of induction.

The calculated values of irreversible permeability *µ_irrev_* as a function of magnetic induction *B* for both compacted samples are shown in Figure 7.

Unexpectedly, higher values of irreversible permeability are achieved for sample S which has fewer obstacles for domain-wall displacement in comparison with sample N. On the other hand, irreversible displacements of the domain walls are performed on a background of higher reversible permeability, while displacements of the domain walls for sample S probably occur across larger distances compared to displacements of the domain walls in sample N; moreover, the magnetic induction changes with larger jumps, causing an increased value of irreversible permeability.

### 3.5. Magnetic Barkhausen Noise

Magnetic Barkhausen noise (MBN) measurements were also carried out for a better and deeper understanding of irreversible processes associated with the motion of DWs. MBN signals were measured via RollScan 350 using MicroScan 600 software. Further measurement conditions are listed in Table 2.

Apart from the filtered MBN signals, the following MBN parameters were also extracted: the effective value of the signal (rms), the number of detected MBN pulses, MBN envelopes, the PP (peak position) that corresponds to the magnetic field in which the MBN envelope attains the maximum, and the FWHM (full width at half maximum) of the MBN envelope.

The effective value of the MBN signal is referred as MBN. The contribution of the background noise produced by the sensor is subtracted using the equations referred to in the previous study [22]. MBN pulse counting is based only on those MBN pulses exceeding the background threshold of 40 mV. The MBN parameters were obtained from eight repetitive MBN bursts (four hysteresis cycles).

**Figure 8:**
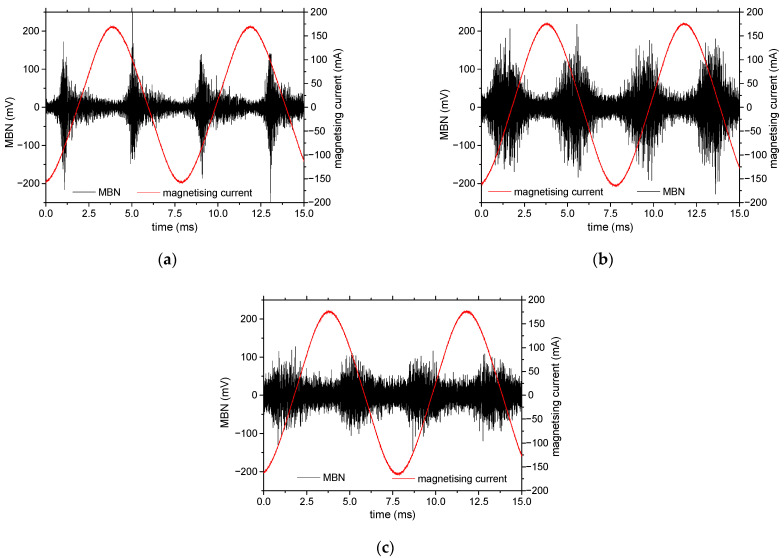
Filtered MBN signals: (**a**) MBN signal for initial sheet, (**b**) MBN signal for sample N, (**c**) MBN signal for sample S.

It should be mentioned that the MBN for all samples is quite low (see Figure 8 and Table 2). MBN is mainly driven by the rate of magnetization change, which is usually linked with DWs’ energy, DWs’ thickness, their density, and the velocity of their motion. However, the energy of magnetocrystalline anisotropy as well as the magnetoelastic energy of the investigated body are low, since the magnetocrystalline anisotropy constant, as well as the isotropic magnetostriction, are close to zero. For this reason, the DW’s energy is very low, its thickness is very wide, and the local misorientation of the neighboring atoms within the DW’s thickness is also very low.

The MBN for the non-smoothed powder is remarkably greater than that produced by the initial sheet and/or the smoothed powder. The effective (rms) value of the MBN depends on the number of MBN pulses *n* and their magnitude *X_i_* as follows:

(5) $$rms=\sqrt{\frac{1}{n}\overset{n}{\underset{i=1}{\sum }}X_{i}^{2}}$$

**Figure 9:**
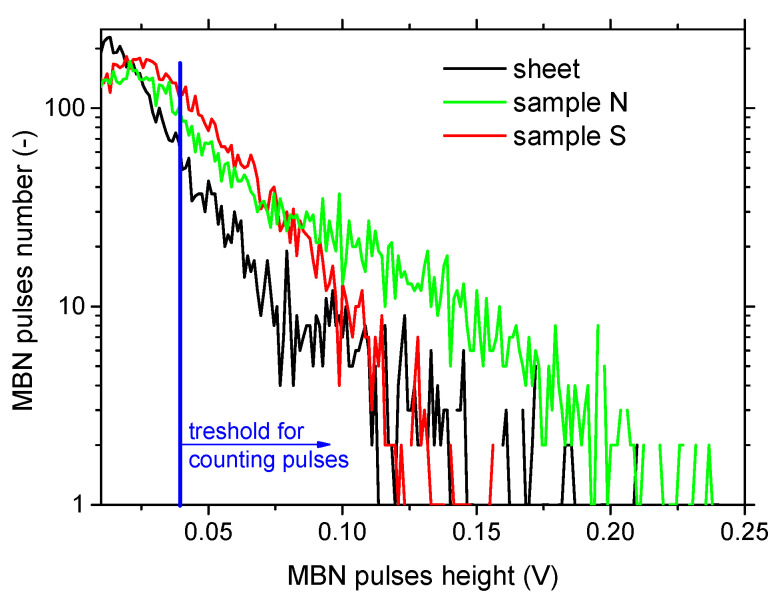
MBN pulse height distribution for initial sheet, sample N, and sample S.

Figure 9 and Table 2 indicate that the higher MBN for sample N is mainly due to the higher number of strong pulses. Figure 9 clearly depicts that MBN pulses of the height exceeding 0.17 V can be detected for sample N in contrast with sample S. This comparison is reversed for the weaker pulses. It should be noted that the number of detected pulses may not be directly linked with the number of movable DWs due to the clustering effect [23] and overlapping of the electromagnetic pulses produced by the individual DW in time.

**Figure 10:**
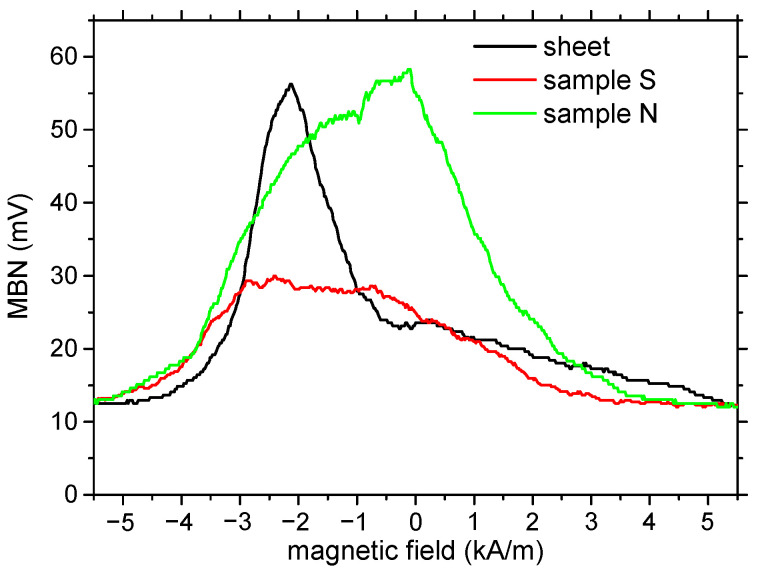
MBN envelopes for initial sheet, sample N, and sample S.

It should be considered that the stronger MBN for the non-smoothed powder is due to sharp edges that act as pinning sites for DWs. The MBN envelopes presented in Figure 10 also prove that the MBN for sample N is stronger. Moreover, the higher pinning strength of the edges delays the irreversible DW process in time and the corresponding magnetic field. The PP values (PP refers to the value of the magnetic field in which an MBN envelope attains a maximum) extracted from the MBN envelope for sample N is found to be closer to zero (see Figure 10, Table 3 and Table 4). The extracted PP values correlate with coercivity (Table 1).

The fragmentation of the solid body into powder remarkably increases the free surface, which can be linked with the increasing density of the DWs, especially those at 90°, as well as the refinement of 180° DWs. The MBN envelopes, as well as FWHM (see Table 3), indicate that fragmentation widens the range of the magnetic field in which the major activity of irreversible DW motion occurs. Increasing density of finer DWs also proves that E90 and E180 energies are extracted from MBN envelopes, as illustrated in Figure 10, when the simplified model of Martinez-Ortiz et al. [24] is employed. E90 is usually linked with the motion of 90° DWs (the contribution of fine 180° DWs should also be considered), whereas E180 is obtained from the MBN envelope near PP (mainly attributed to the motion of large 180° DWs). E90 for sample S is greater than that for the solid body at the expense of reduced E180. On the other hand, the sharp edges on the non-smoothed powder contribute to higher E90 and E180. In other words, the conditions in which DWs are unpinned are less homogenous for the powder samples in contrast with the solid body (sheet).

### 3.6. Losses

The analysis of core losses supports the evidence of the negative influence of surface irregularities on domain-wall displacement.

The separation of the core losses leads to the sum of three components (hysteresis *W_dc_*, the classical eddy current *W_cl_*, and excess losses *W_ex_* [19,25]), which is well known according to Bertotti’s statistical model [26,27,28].

The core energy losses *W_dc_* (J/m^3^) are then expressed as follows:

(6) $$W_{c}=W_{dc}+W_{cl}+W_{ex}=C_{dc}+C_{cl}f+C_{ex}f^{1/2}$$

where the coefficients *C_dc_* (equal to hysteresis loss), *C_cl_*, and *C_ex_* were calculated by fitting the experimental results in [7,10].

**Figure 11:**
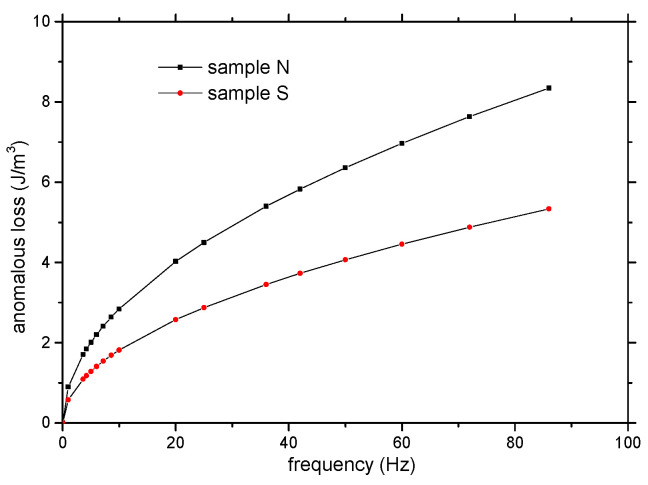
Excess loss of sample N and sample S as a function of frequency.

The frequency dependences of excess loss separated from the core energy loss of both samples measured for *B_m_* = 0.2 T are in Figure 11, plotted as a third addendum in Equation (6).

The details regarding hysteresis and classical eddy current excess loss are described in [10,15].

(7) $$W_{ex}=\frac{8\rho B_{m}}{n_{0}}{\left(\frac{GSB_{m}}{V_{0}\rho_{R}}\right)}^{\frac{1}{2}}f^{\frac{1}{2}},$$

where *ρ* is the density of the material, *ρ_R_* is the resistivity of the material, *B_m_* is the maximum induction, *G* is the dimensionless coefficient, *V*_0_ is the material parameter, *S* is the cross-section of the ring sample, and *n*_0_ is the number of movable magnetic objects (domain walls) in the cross-section of the measured material in the shape of a ring. The coefficient *C_a_*,

(8) $$C_{ex}=\frac{8\rho B_{m}}{n_{0}}{\left(\frac{GSB_{m}}{V_{0}\rho_{R}}\right)}^{\frac{1}{2}}\sim \frac{1}{n_{0}},$$

is inversely proportional to *n*_0_.

The numeric values of *C_ex_* (calculated for *B_m_* = 0.2 T) were published in [10] and are presented in Table 1. If we suppose that the parameters in (8) are not too different for both samples, sample S (prepared from particles with smoothed surfaces) has more movable domain walls in the cross-section of the ring in comparison with sample N (prepared from powder particles with non-smoothed surfaces). When a significant fraction of the domain walls are non-movable, they are “pinned” on the surface irregularities of powder particles.

## 4. Conclusions

The influence of mechanical smoothing applied to Ni80Fe15Mo5 (wt.%) alloy particle surfaces before compaction was investigated using different experimental methods such as coercive field, permeability, excess loss, and Barkhausen noise analysis. The compaction prepared from smoothed powder particles (sample S) exhibits better soft magnetic properties (lower coercivity, higher initial and maximum relative permeability, and low-frequency core losses) in comparison with the compaction prepared under the same conditions from non-smoothed particles (sample N). The positive influence of the smoothing process applied to particle surfaces on the soft magnetic properties was explained by the reduction in the number of surface obstacles for domain-wall displacement, and was detected as follows:

1. The irregular shape of the powder particles of sample N leads to better contact between the particles, but magnetization processes take place more easily in the particles of sample S, where the capture centers for the displacement of the domain walls are reduced.
2. There is a lower coercive field in the frequency range from DC to 1 kHz at the maximum induction of 0.2 T for sample S in comparison with sample N.
3. There are higher values of differential relative permeability and reversible relative permeability in magnetization reversal in the DC magnetic field at the maximum induction of 0.2 T for sample S in comparison with sample N,
4. The lower values of the amplitude of the Barkhausen noise for sample S in comparison with sample N confirms the assumption that a larger number of easily movable domain walls are present over shorter distances in sample S.
5. There is lower excess loss in the frequency range from DC to 1 kHz measured at the maximum induction of 0.2 T for sample S in comparison with sample N.

## Figures and Tables

**Table 1:** The parameters of both samples.

Sample	Density ^a^ g/cm^3^	Coercivity ^a^ A/m	DC Losses ^b^ J/m^−3^	Initial Relative Permeability ^a^	Maximum Relative Permeability ^a^	Excess Loss Coefficient ^b^ J·m^−3^·s^1/2^
N	7.30	24.4	4.60	3400	8480	0.90
S	7.15	4.80	1.54	10,850	27,700	0.58

^a^ Originally published in [8]; ^b^ originally published in [10].

**Table 2:** Experimental conditions for measurements of MBN.

Conditions of MBN	Values
Magnetizing frequency	125 Hz of sine profile
Magnetizing voltage	6 V
Sensor	S1-18-12-01
MBN signal frequency range	From 10 to 150 kHz
Sampling frequency	6.7 MHz

**Table 3:** MBN parameters.

Sample	Rms mV	Number of MBN Pulses	PP kA·m^−1^	FWHM kA·m^−1^
Sheet	23.5 ± 2.5	2232 ± 160	−2.16 ± 0.01	1.35 ± 0.07
N	38.5 ± 3.5	5521 ± 267	−0.65 ± 0.03	4.05 ± 0.11
S	25.0 ± 3.0	2920 ± 150	−2.21 ± 0.01	4.40 ± 0.06

**Table 4:** MBN energies extracted from MBN envelopes.

Sample	E^90^ (mV)^2^	E^180^ (mV)^2^
Sheet	4	68
N	54	85
S	16	35

## Data Availability

The data presented in this study are available from the corresponding authors upon reasonable request.
